# Interpretable machine learning to predict NOAF in ICU patients with CKD: validation in US and Chinese cohorts

**DOI:** 10.3389/fmed.2026.1816221

**Published:** 2026-05-13

**Authors:** Shujie Zhang, Na Ren, Qing Yin, Chao Yang, Lujing Nie, Jianan Zhao, Yuxiu Liu, Jing Huang, Yanbo Chen

**Affiliations:** 1 Clinical Medical School, Shandong Second Medical University, Weifang, Shandong, China; 2 Department of Cardiology, Weifang People’s Hospital, Shandong Second Medical University, Weifang, Shandong, China; 3 Department of Vascular Surgery, Weifang People’s Hospital, Shandong Second Medical University, Weifang, Shandong, China; 4 Department of Critical Care Medicine, Fuzhou University Provincial Affiliated Hospital, Fuzhou, Fujian, China; 5 School of Nursing, Shandong Second Medical University, Weifang, Shandong, China

**Keywords:** atrial fibrillation, CKD, eICU-CRD, machine learning, MIMIC

## Abstract

**Objective:**

Critically ill patients with chronic kidney disease (CKD) are at high risk for New-Onset Atrial Fibrillation (NOAF), which significantly increases their risk of adverse events. Early detection of NOAF is crucial for prompt intervention and better outcomes. This study aims to develop and validate predictive models for the early identification and stratification of NOAF risk in this vulnerable population.

**Methods:**

We developed and validated a predictive model using a cohort of 6,476 critically ill patients with CKD from the Medical Information Mart for Intensive Care-IV (MIMIC-IV) database. After performing feature selection via least absolute shrinkage and selection operator (Lasso) logistic regression, we trained six machine learning (ML) models. These algorithms included: Random Forest, Gradient Boosting, eXtreme Gradient Boosting (XGBoost), Logistic Regression (LR), Multi-layer Perceptron (MLP), and Light Gradient Boosting Machine (LightGBM). The best-performing model was interpreted using SHAP to provide clinical insights. Its robustness and generalizability were confirmed through rigorous external validation on two distinct international cohorts: the US-based eICU-CRD (eICU Collaborative Research Database) (*n* = 12,509) and a Chinese ICU database from Weifang People’s Hospital (*n* = 880).

**Results:**

Ultimately, 12 predictive features were ultimately selected: age, SOFA score, minimum heart rate, congestive heart failure, average heart rate, minimum systolic blood pressure (SBP), mechanical ventilation use, minimum oxygen saturation (SpO_2_), average respiratory rate, minimum magnesium, SAPS II score, and maximum white blood cell (WBC) count. The Random Forest model demonstrated the best overall performance, achieving an area under the receiver operating characteristic curve (AUC) of 0.855 in internal validation. The model’s robustness was confirmed through external validation on two independent cohorts, yielding an AUC of 0.760 on the eICU-CRD and 0.740 on the Weifang People’s Hospital database. According to the SHAP analysis, age, average heart rate, minimum heart rate, SOFA score, and SAPS II were the top five most influential predictors for NOAF.

**Conclusion:**

We developed an interpretable machine learning model to predict NOAF in critically ill CKD patients, demonstrating strong generalizability through external validation on both a large US cohort (eICU-CRD) and a single-center Chinese cohort (Weifang People’s Hospital). SHAP analysis enhanced model interpretability.

## 1 Introduction

NOAF represents the most prevalent new-onset arrhythmia in the intensive care unit (ICU) setting (1). Reported incidence rates of NOAF among ICU patients range from 10 to 40%, constituting a substantial clinical challenge (2). The onset of atrial fibrillation, often precipitated by these stressors, is associated with prolonged ICU stays, increased healthcare expenditure, and adverse clinical outcomes (3). Irregular atrial contractions from AF reduce cardiac output by 20–30%, a hemodynamic compromise especially severe in patients with ventricular systolic dysfunction (4). At last but not least, patients with NOAF are at significantly increased risk for in-hospital mortality (5), cardiogenic shock (6), and worsening renal function (7).

Among well-established independent risk factors for NOAF, CKD stands as a significant contributor (8). Chronic electrolyte disturbances, volume overload, inflammatory responses, and uremic toxin accumulation in CKD settings cause atrial structural and electrophysiological remodeling (9), creating conditions for atrial fibrillation development and producing a 2.18-to 3.2-fold increased risk for NOAF compared to those without CKD (10, 11).

Atrial fibrillation is a prevalent secondary arrhythmia in patients with CKD, occurring at a significantly higher rate than in the general population (12). AF not only serves as a marker of cardiovascular disease but also independently predicts poor prognosis, including increased all-cause and cardiovascular mortality, especially in dialysis patients (3). Early identification of CKD patients at high risk for AF is therefore crucial. Consequently, the early prediction and identification of NOAF in individuals with CKD is crucial for effective risk stratification and enhancing long-term patient outcomes.

At present, the tools available for predicting the occurrence of NOAF in critically ill patients with CKD have significant limitations. Existing risk scores (such as CHARGE-AF) are developed based on the general population and are unable to capture acute and dynamic risk factors specific to the ICU environment, such as the use of vasopressors and severe inflammatory states. On the contrary, the existing NOAF prediction models for ICUs either only cover general ICU patients without focusing on the high-risk CKD subgroup, or lack strong external validation, which limits their clinical applicability.

To fill this key gap, this study mainly makes the following two contributions: Firstly, we developed and conducted external validation, establishing a machine learning model specifically for predicting the occurrence of NOAF in critically ill CKD patients - a population with unique pathophysiological characteristics and risk manifestations. Secondly, our model only requires 12 easily accessible clinical and laboratory variables, aiming to facilitate integration into clinical workflows without relying on special or expensive data support. Finally, we adopted SHapley Additive exPlanations (SHAP) analysis to ensure the interpretability of the model, helping clinicians understand the key drivers of individual patient risks and thus take more confident diagnostic and therapeutic actions.

## 2 Methods

### 2.1 Study design and data source

**Figure 1:**
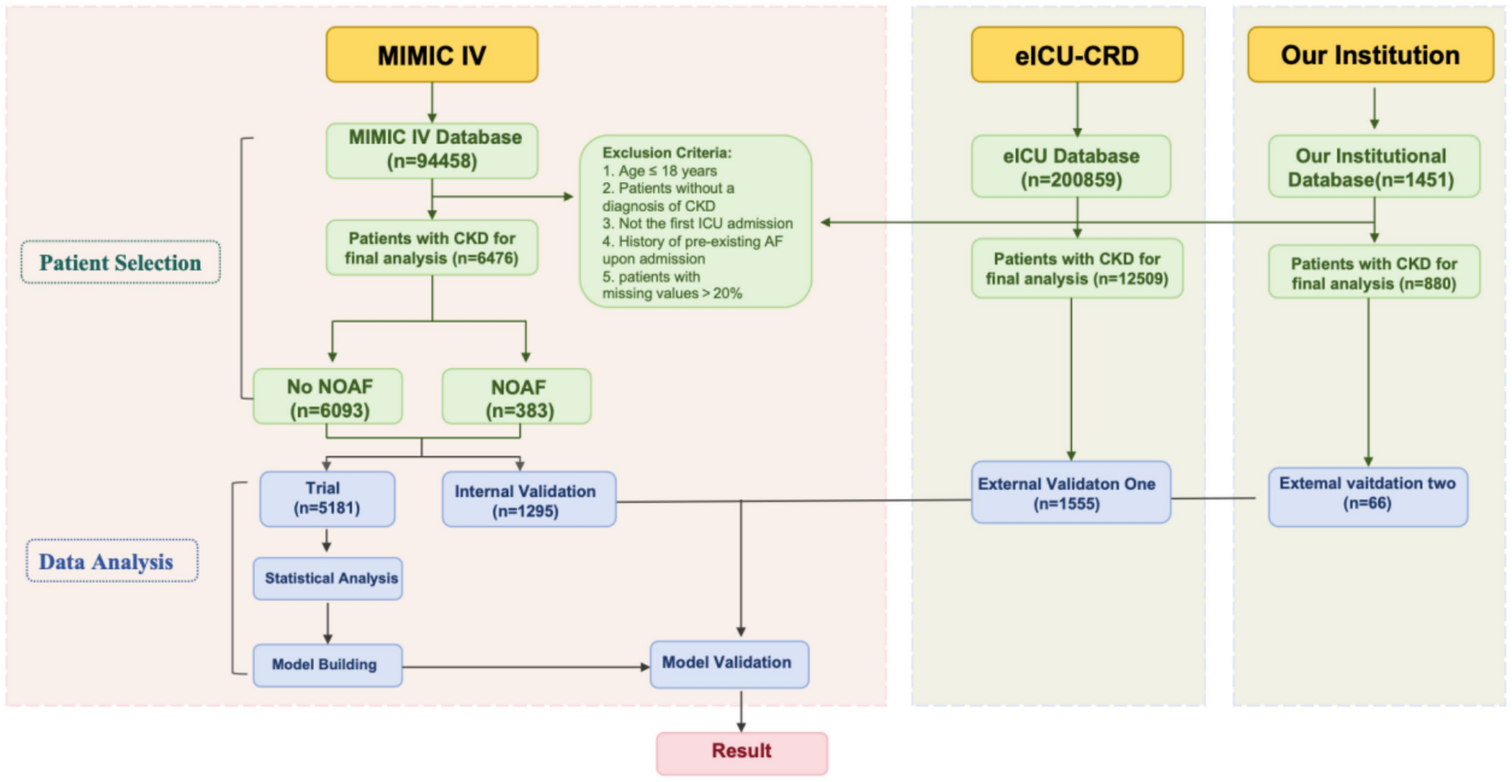
Work flowchart. MIMIC-IV: Medical Information Mart for Intensive Care IV, eICU-CRD: eICU Collaborative Research Database, CKD: chronic kidney disease.

Data for this retrospective cohort study were obtained from three large-scale critical care databases, with the specific extraction workflow detailed in Figure 1. The model development and internal validation cohort was sourced from the Medical Information Mart for Intensive Care IV (MIMIC-IV, v2.2), which includes high-resolution, de-identified clinical data from intensive care unit patients at the Beth Israel Deaconess Medical Center between 2008 and 2019. To assess the model’s generalizability, external validation was performed on two distinct and independent cohorts. The first was the eICU Collaborative Research Database (eICU-CRD, v2.0), a multi-center US database from 208 hospitals (2014–2015). The second was a single-center database collected from the intensive care unit of Weifang People’s Hospital in China, covering the period from 2017 to 2025, allowing for a comprehensive evaluation of the model’s performance across different healthcare systems, care settings, and patient populations. The author Chao Yang completed the required CITI training certification (Record ID: 12473044). This study was conducted in accordance with the Declaration of Helsinki and was approved by the Ethics Committee of Weifang People’s Hospital (Approval Number: KYLI20241008-20). As this study was a retrospective one and involved de-identified data, the committee waived the requirement for informed consent.

### 2.2 Cohort selection

Study participants were selected from the MIMIC-IV database, focusing on adult patients with CKD experiencing their first ICU admission, while excluding those with pre-existing atrial fibrillation, individuals under 18 years of age, or cases where key variables had more than 20% missing data.

NOAF is defined as the first recorded episode of atrial fibrillation during hospitalization in the intensive care unit. To standardize the timing of diagnosis across all datasets, the observation period began exactly at the timestamp of ICU admission (T0). The time of NOAF onset was standardized as the recorded timestamp of the first positive electrocardiogram (ECG) report or clinical note confirming the arrhythmia after T0.

We identified atrial fibrillation events through the codes of ICD-9 (427.31) and ICD-10 (I48.x) recorded in the patients’ medical records. To ensure that they are truly new cases, we excluded patients who had any diagnostic records of atrial fibrillation or atrial flutter before being admitted to the ICU this time. To ensure diagnostic accuracy and address potential inaccuracies in disease coding, all initial NOAF diagnoses based on ICD codes were cross-validated with free-text electrocardiogram (ECG) reports by identifying terms such as “atrial fibrillation” or “AF.” For large public databases (MIMIC-IV and eICU-CRD), this cross-validation included automated text mining, followed by manual review of all positive results and discrepancies. In cases of data conflict—such as patients with an ICD code for NOAF but all ECG reports showing normal sinus rhythm, or the opposite scenario—the clinical ECG text reports were strictly regarded as the ultimate gold standard. For the Weifang People’s Hospital cohort, 100% manual confirmation of all NOAF cases and their corresponding ECGs was performed by clinicians.

This process resulted in a development cohort of 6,476 patients, among whom 383 developed NOAF during their ICU stay. Applying the same inclusion, exclusion, and adjudication criteria yielded two distinct external validation cohorts. The first, derived from the eICU-CRD, comprised 12,509 patients with 1,555 observed cases of NOAF. The second cohort, from the Weifang People’s Hospital ICU database, consisted of 880 patients, among whom 66 developed NOAF.

### 2.3 Variable extraction and feature selection

Patient data were extracted through PostgreSQL, with time-varying vital signs and laboratory parameters captured as maximum, minimum, and mean values during the observation period. Variables covered six categories: baseline characteristics such as age, gender, ethnicity, insurance type, language, and admission source; clinical severity scores and hospitalization details including SOFA, SAPS-II, Charlson Comorbidity Index, hospital and ICU length of stay; comorbidities derived from Charlson components such as myocardial infarction, congestive heart failure, peripheral vascular disease, cerebrovascular disease, dementia, chronic pulmonary disease, rheumatic disease, peptic ulcer disease, liver disease, diabetes, renal disease, cancer, and AIDS; vital signs covering heart rate, systolic blood pressure, respiratory rate, temperature, and SpO_2_; laboratory parameters including electrolytes, renal function markers, hematological indices, and coagulation profiles; and interventions like vasopressor use, mechanical ventilation, and renal replacement therapy, with NOAF serving as the primary outcome.

All features were extracted within the first 24 h of the patient’s admission to the ICU to ensure that the model could provide early prediction. For time-varying vital signs (such as heart rate and blood pressure) and laboratory test results, we calculated the maximum, minimum and average values within this 24-h window. Baseline characteristics such as age and comorbidities were extracted from the admission records. The SOFA and SAPS-II scores were calculated based on the values at admission.

We performed variable selection using backward stepwise elimination in a multivariable logistic regression model. Variables were iteratively removed from an initial full model if their *p*-value was greater than 0.05, until all remaining predictors were statistically significant. This process yielded a final set of 12 predictors for model construction. The importance of these variables, ranked by the absolute values of their coefficients, is shown in Figure 1.

### 2.4 Data preprocessing

Before model training, we implemented a strict data preprocessing procedure to ensure data quality. Firstly, variables with a missing rate exceeding 20% were completely excluded from the study. Secondly, for common measurement artifacts or recording errors in electronic health records of intensive care units, we applied an outlier management strategy. Physiologically implausible extreme values (such as obvious outliers in vital signs or laboratory tests) were identified as outliers and strictly converted to missing values (NA). These converted missing values, along with the original missing data, were then processed together in our unified imputation procedure.

### 2.5 Missing value handling

To address the issue of data missingness, this study employed a hierarchical imputation approach. Variables with a missing rate below 5% were imputed using the mean imputation method; variables with a missing rate between 5 and 20% were processed using the Multiple Imputation by Chained Equations (MICE) algorithm, specifically utilizing the predictive mean matching (PMM) method to preserve the original distribution of the clinical features. The variables requiring multiple imputation included: peak international normalized ratio (INR) (missing rate 18.3%), peak partial thromboplastin time (PTT) (missing rate 17.6%), lowest blood calcium level (missing rate 6.5%), and highest blood phosphorus level (missing rate 5.5%). The missing rate for all other variables was less than 2%.

### 2.6 Statistical analysis and model development

Descriptive statistics presented continuous variables as mean ± standard deviation and categorical variables as counts with percentages, while group comparisons for continuous variables utilized Welch’s t-test or ANOVA based on homogeneity of variance, and categorical variable comparisons employed the Chi-squared test, with Fisher’s exact test applied when expected cell counts fell below five.

The development cohort from MIMIC-IV was divided randomly into training and testing subsets at an 8:2 ratio. To address structural class imbalance while rigorously preventing data over-optimism, the Synthetic Minority Over-sampling Technique (SMOTE) was utilized and strictly embedded within the cross-validation pipeline to avoid data leakage. Specifically, six machine learning approaches (RF, GB, XGBoost, LR, MLP, and LightGBM) were trained using 10-fold cross-validation. During each iteration of the cross-validation, SMOTE was exclusively restricted to the internal training fold, generating synthetic minority samples to create a 1:1 balanced distribution for model fitting. Conversely, all corresponding internal validation folds, as well as the independent 20% test subset and external validation cohorts, remained entirely untouched by the oversampling algorithm. These hold-out sets preserved their natural, unmodified imbalanced status, ensuring that the model’s true discrimination and generalizability were honestly evaluated in real-world clinical distributions. The top-performing model was ultimately selected for extensive evaluation and external validation.

During the 10-fold cross-validation process, hyperparameter tuning was conducted using Grid Search to optimize model performance, with the Area under the Receiver Operating Characteristic Curve (AUC) serving as the primary evaluation metric. For the top-performing Random Forest model, the hyperparameter search space incorporated a comprehensive range of architectural configurations, systematically exploring various combinations of the total number of trees (n_estimators), the maximum depth of the trees (max_depth), and the thresholds for minimum samples required for broad internal node splitting (min_samples_split) and terminal leaf nodes (min_samples_leaf).

### 2.7 Model evaluation

Model performance was evaluated using a comprehensive set of standard metrics. Discrimination was quantified by the AUC, accuracy, sensitivity, specificity, and F1 score. Calibration was assessed through calibration curves and the Hosmer-Lemeshow test to evaluate agreement between predicted and observed risks. Beyond traditional statistical measures, we used DCA to assess clinical utility and net benefit for decision-making. To enhance interpretability, we performed SHAP analysis on the best-performing model to characterize the impact of each predictor on individual outcomes.

### 2.8 External validation and data harmonization

To evaluate the generalization ability and robustness of the final model, we conducted external validation using two independent cohorts: a multicenter public database, the eICU-CRD (v2.0), and a single-center, in-house database from the ICU of Weifang People’s Hospital. For both validation cohorts, we applied the same inclusion and exclusion criteria as the MIMIC-IV development cohort.

To ensure a fair evaluation of the model in the external eICU-CRD cohort, we implemented a strict data standardization process. Firstly, we mapped the variables between the MIMIC-IV and eICU-CRD because they use different annotation systems (for example, mapping eICU’s “sao_2_” to MIMIC-IV’s “SpO_2_”). Secondly, all laboratory test values are converted to uniform measurement units (for example, ensuring that all creatinine values are in mg/dL units).

To maintain strict methodological consistency and ensure independent reproducibility, we applied the exact same data preprocessing pipeline to the external validation cohorts (eICU-CRD and Weifang People’s Hospital cohort) as that used in the MIMIC-IV development cohort. Specifically, both validation cohorts adopted the identical outlier handling strategy (converting physiologically implausible extreme values to missing data) and the same hierarchical missing data processing procedure (excluding variables with a missing rate greater than 20%, imputing variables with a missing rate below 5% using mean imputation, and applying the MICE method for multiple imputation to variables with a missing rate between 5 and 20%). By keeping the feature extraction time window and preprocessing parameters completely symmetrical across all datasets, we ensured a fair and unbiased assessment of the model’s true generalization ability.

## 3 Results

### 3.1 Patient baseline characteristics

The training cohort was derived from the MIMIC-IV database (n = 6,475). For external validation, we utilized two independent cohorts: the multi-center eICU-CRD (*n* = 12,509; NOAF incidence: 12.4%) and our single-center institutional cohort (*n* = 880; NOAF incidence: 7.5%).

Table 1 presents the baseline demographic and clinical characteristics for patients in both the MIMIC-IV and eICU-CRD cohorts, stratified by the development of NOAF. In the MIMIC-IV cohort (*N* = 6,476), patients who developed NOAF (*n* = 383) were significantly older (75 ± 12 vs. 68 ± 15 years, *p* < 0.001), had longer hospital and ICU stays, and presented with a greater severity of illness, as evidenced by higher Charlson Comorbidity Index, SOFA, and SAPS II scores(all *p* < 0.001). Clinically, the NOAF group exhibited signs of greater instability, including lower minimum SBP (82 ± 20 vs. 94 ± 20 mmHg) and minimum SpO_2_ (81 ± 17 vs. 89 ± 10%), alongside a higher average respiratory rate (all *p* < 0.001). Laboratory analyses revealed that these patients had significantly lower minimum levels of bicarbonate, hemoglobin, and platelets, but higher maximum levels of BUN, WBC, PTT, and INR, reflecting a more severe systemic dysfunction. Similar trends were observed in the eICU-CRD cohort (*N* = 12,509). NOAF patients (*n* = 1, 640) were significantly older (74 ± 12 vs. 66 ± 15 years, *p* < 0.001), experienced prolonged hospitalizations, and had higher illness severity scores (SAPS II: 73 ± 26 vs. 63 ± 25, *p* < 0.001). In this cohort, the proportion of male patients was also significantly higher in the NOAF group (60.3%vs. 55.1%, *p* < 0.001). Vital sign abnormalities were prominent, with the NOAF group demonstrating higher average and maximum heart rates, a higher average respiratory rate, and lower minimum SBP and SpO2 (all p < 0.001). Key laboratory differences included higher maximum levels of WBC and PTT, but contrary to the MIMIC-IV cohort, significantly lower maximum creatinine levels in the NOAF group (p < 0.001).

**Table 1:** Patient demographics and baseline characteristics.

Characteristic	MIMIC IV, *N* = 6,476				eICU-CRD, *N* = 12,509			
	Overall *N* = 6,476	Non-NOAF *N* = 6,093	NOAF *N* = 383	*p*-value^1^	Overall *N* = 12,509	Non-NOAF *N* = 10,869	NOAF *N* = 1,640	p-value^1^
Age, Mean ± SD	68 ± 15	68 ± 15	75 ± 12	<0.001	67 ± 15	66 ± 15	74 ± 12	<0.001
Gender, *n* (%)				0.177				<0.001
Male	3,941 (60.9%)	3,695 (60.6%)	246 (64.2%)		6,981 (55.8%)	5,992 (55.1%)	989 (60.3%)	
Female	2,535 (39.1%)	2,398 (39.4%)	137 (35.8%)		5,525 (44.2%)	4,875 (44.9%)	650 (39.7%)	
Hospital_los, Mean ± SD	11 ± 12	11 ± 12	14 ± 15	<0.001	8 ± 10	8 ± 9	11 ± 17	<0.001
Icu_los, Mean ± SD	3.5 ± 5.1	3.2 ± 4.6	7.1 ± 9.5	<0.001	3.5 ± 6.7	3.2 ± 4.5	5.7 ± 14.4	<0.001
Charlson_comorbidity_index, Mean ± SD	7.62 ± 2.54	7.56 ± 2.54	8.48 ± 2.31	<0.001	3.47 ± 1.54	3.39 ± 1.50	4.02 ± 1.63	<0.001
Sofa_score, Mean ± SD	6.0 ± 3.6	5.8 ± 3.5	8.8 ± 4.5	<0.001	2.62 ± 1.90	2.62 ± 1.91	2.65 ± 1.84	0.490
Sapsii, Mean ± SD	40 ± 14	40 ± 13	50 ± 15	<0.001	64 ± 26	63 ± 25	73 ± 26	<0.001
Heart_rate_avg, Mean ± SD	81 ± 13	81 ± 13	84 ± 13	<0.001	83 ± 16	82 ± 15	87 ± 18	<0.001
Heart_rate_min, Mean ± SD	63 ± 17	63 ± 17	53 ± 23	<0.001	68 ± 15	68 ± 15	71 ± 16	<0.001
Heart_rate_max, Mean ± SD	105 ± 90	104 ± 92	122 ± 27	<0.001	105 ± 22	103 ± 21	113 ± 25	<0.001
sbp_min, Mean ± SD	94 ± 20	94 ± 20	82 ± 20	<0.001	88 ± 23	89 ± 23	82 ± 20	<0.001
sbp_max, Mean ± SD	183 ± 17	185 ± 18	156 ± 26	0.223	163 ± 30	164 ± 30	156 ± 28	<0.001
resp_rate_avg, Mean ± SD	19.0 ± 3.4	18.9 ± 3.4	20.2 ± 3.4	<0.001	19.3 ± 4.4	19.2 ± 4.4	20.1 ± 4.3	<0.001
resp_rate_min, Mean ± SD	9.9 ± 4.8	10.0 ± 4.7	8.1 ± 5.5	<0.001	11.2 ± 4.1	11.1 ± 4.1	11.9 ± 4.3	<0.001
resp_rate_max, Mean ± SD	32 ± 50	32 ± 52	36 ± 15	<0.001	31 ± 9	31 ± 9	31 ± 8	0.477
temp_c_avg, Mean ± SD	36.8 ± 0.1	36.8 ± 0.2	36.7 ± 0.9	<0.001	36.7 ± 0.6	36.7 ± 0.6	36.7 ± 0.7	0.684
temp_c_min, Mean ± SD	36.0 ± 0.3	36.0 ± 0.5	35.2 ± 0.7	<0.001	36.2 ± 0.8	36.2 ± 0.8	36.2 ± 0.9	0.125
temp_c_max, Mean ± SD	38.6 ± 0.6	38.5 ± 0.7	39.0 ± 0.6	<0.001	37.2 ± 0.7	37.2 ± 0.7	37.2 ± 0.8	0.642
SpO_2__min, Mean ± SD	88 ± 11	89 ± 10	81 ± 17	<0.001	85 ± 10	86 ± 10	83 ± 11	<0.001
Potassium_min, Mean ± SD	4.2 ± 0.7	4.2 ± 0.7	4.2 ± 0.7	0.202	4.2 ± 0.7	4.2 ± 0.7	4.2 ± 0.7	0.472
Potassium_max, Mean ± SD	4.7 ± 0.9	4.7 ± 0.9	4.9 ± 0.9	<0.001	4.5 ± 0.8	4.5 ± 0.8	4.5 ± 0.8	0.686
Sodium_min, Mean ± SD	137.1 ± 5.3	137.1 ± 5.3	137.0 ± 5.4	0.559	137.0 ± 5.2	137.0 ± 5.1	137.2 ± 5.5	0.208
Sodium_max, Mean ± SD	139.3 ± 5.2	139.3 ± 5.2	139.7 ± 5.6	0.177	138.4 ± 5.1	138.4 ± 5.0	138.6 ± 5.4	0.176
Chloride_min, Mean ± SD	102 ± 7	102 ± 7	102 ± 7	0.767	102 ± 7	102 ± 7	102 ± 7	0.768
Chloride_max, Mean ± SD	105 ± 7	105 ± 7	105 ± 7	0.244	104 ± 7	104 ± 7	104 ± 7	0.504
Bicarbonate_min, Mean ± SD	21.0 ± 4.9	21.1 ± 4.9	19.6 ± 5.0	<0.001	23.2 ± 5.4	23.2 ± 5.4	23.3 ± 5.6	0.294
Bicarbonate_max, Mean ± SD	23.0 ± 4.4	23.1 ± 4.4	22.3 ± 4.5	0.002	24.5 ± 4.9	24.5 ± 4.8	24.8 ± 5.1	0.016
Creatinine_min, Mean ± SD	127 ± 54	127 ± 54	132 ± 57	0.135	123 ± 53	124 ± 53	133 ± 62	<0.001
Creatinine_max, Mean ± SD	173 ± 116	172 ± 117	182 ± 101	0.063	174 ± 95	176 ± 113	192 ± 103	<0.001
Bun_max, Mean ± SD	45 ± 29	44 ± 29	50 ± 31	<0.001	45 ± 28	45 ± 28	48 ± 27	<0.001
Magnesium_min, Mean ± SD	2.0 ± 0.5	2.0 ± 0.4	2.1 ± 0.6	0.002	2.0 ± 0.4	2.0 ± 0.4	2.0 ± 0.4	0.648
Calcium_min, Mean ± SD	8.3 ± 0.9	8.3 ± 0.9	8.1 ± 0.9	<0.001	8.3 ± 0.9	8.3 ± 0.9	8.3 ± 0.9	0.067
Phosphate_max, Mean ± SD	4.6 ± 1.8	4.5 ± 1.8	5.1 ± 2.2	<0.001	4.7 ± 2.0	4.7 ± 2.0	4.5 ± 1.8	0.007
Hematocrit_min, Mean ± SD	29 ± 6	29 ± 6	29 ± 7	0.028	30 ± 6	30 ± 6	31 ± 6	0.003
Hemoglobin_min, Mean ± SD	9.6 ± 2.1	9.6 ± 2.1	9.3 ± 2.1	0.009	9.9 ± 2.1	9.9 ± 2.1	10.0 ± 2.1	0.030
Platelet_min, Mean ± SD	185 ± 95	186 ± 96	164 ± 91	<0.001	186 ± 88	187 ± 87	181 ± 94	0.028
wbc_max, Mean ± SD	13 ± 14	13 ± 14	17 ± 23	<0.001	12 ± 11	12 ± 11	13 ± 9	<0.001
ptt_max, Mean ± SD	44 ± 31	44 ± 31	52 ± 37	<0.001	46 ± 29	45 ± 28	49 ± 31	0.002

^1^Welch Two Sample *t*-test; Fisher’s exact test. SD, Standard Deviation; sbp, systolic blood pressure; resp., respiratory rate; temp, temperature; bun, blood urea nitrogen; wbc, white blood cell count; ptt, partial thromboplastin time.

**Table 2:** Performances of the machine learning models for predicting NOAF.

Model	AUC value	Accuracy	Precision	AP	F1
Random forest	0.855	0.856	1.000	0.755	0.869
Gradient boosting	0.847	0.837	0.881	0.716	0.819
XGBoost	0.842	0.858	1.000	0.745	0.679
Logistic regression	0.808	0.763	0.951	0.573	0.615
MLP	0.814	0.836	0.895	0.678	0.710
LightGBM	0.845	0.859	1.000	0.746	0.782

XGBoost, eXtreme Gradient Boosting; MLP, Multi-layer Perceptron; MLP, Multilayer Perceptron; LightGBM, Light Gradient Boosting Machine.

### 3.2 Feature selection results

**Figure 2:**
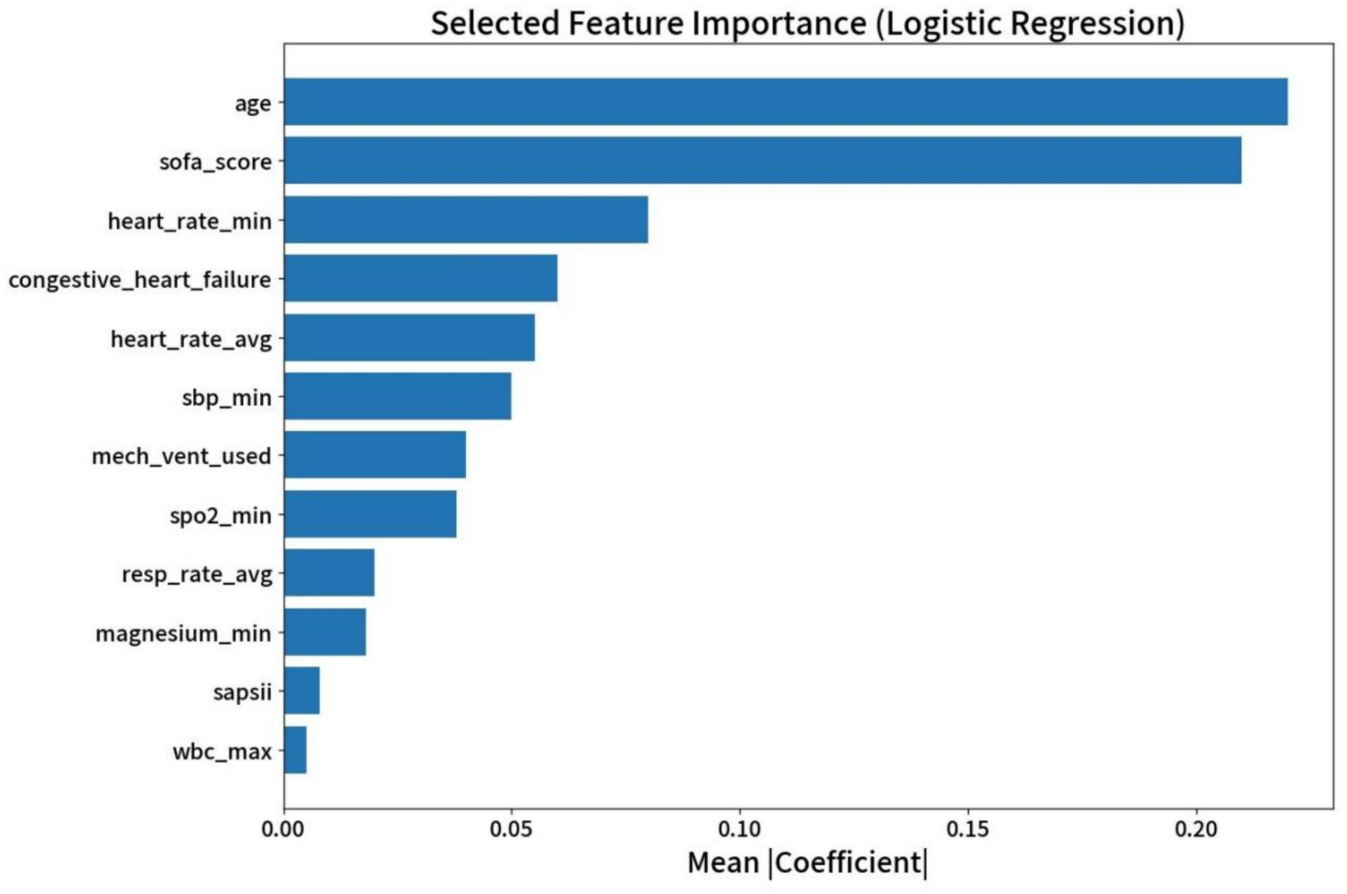
Ranking of feature importance derived from the logistic regression model. sofa_score, sequential organ failure assessment score; heart_rate_min, minimum heart rate; congestive_heart_failure, congestive heart failure; heart_rate_avg, average heart rate; sbp_min, minimum systolic blood pressure; mech_vent_used, mechanical ventilation used; spo2_min, minimum oxygen saturation; resp_rate_avg, average respiratory rate; magnesium_min, minimum magnesium level; sapsii, simplified acute physiology Score ii; wbc_max, maximum white blood cell count.

Following the backward stepwise elimination process in the multivariable logistic regression model, a final set of 12 predictors was identified as being statistically significant (*p* < 0.05). These features were subsequently used for the final model construction. The selected predictors include a combination of demographic data, clinical severity scores, vital signs, comorbidities, interventions, and laboratory values. The relative importance of each feature, quantified by the mean absolute coefficient from the logistic regression model, is presented in Figure 2.

As illustrated, age and SOFA score emerged as the two most powerful predictors in the model. These were followed by physiological variables such as minimum heart rate (heart_rate_min), the presence of congestive heart failure, and average heart rate (heart_rate_avg). Other significant predictors, in descending order of importance, included minimum systolic blood pressure (sbp_min), use of mechanical ventilation (mech_vent_used), minimum SpO_2_ (SpO_2__min), average respiratory rate (resp_rate_avg), minimum magnesium (magnesium_min), SAPS II score, and maximum white blood cell count (wbc_max).

### 3.3 Model performance comparisons

**Figure 3:**
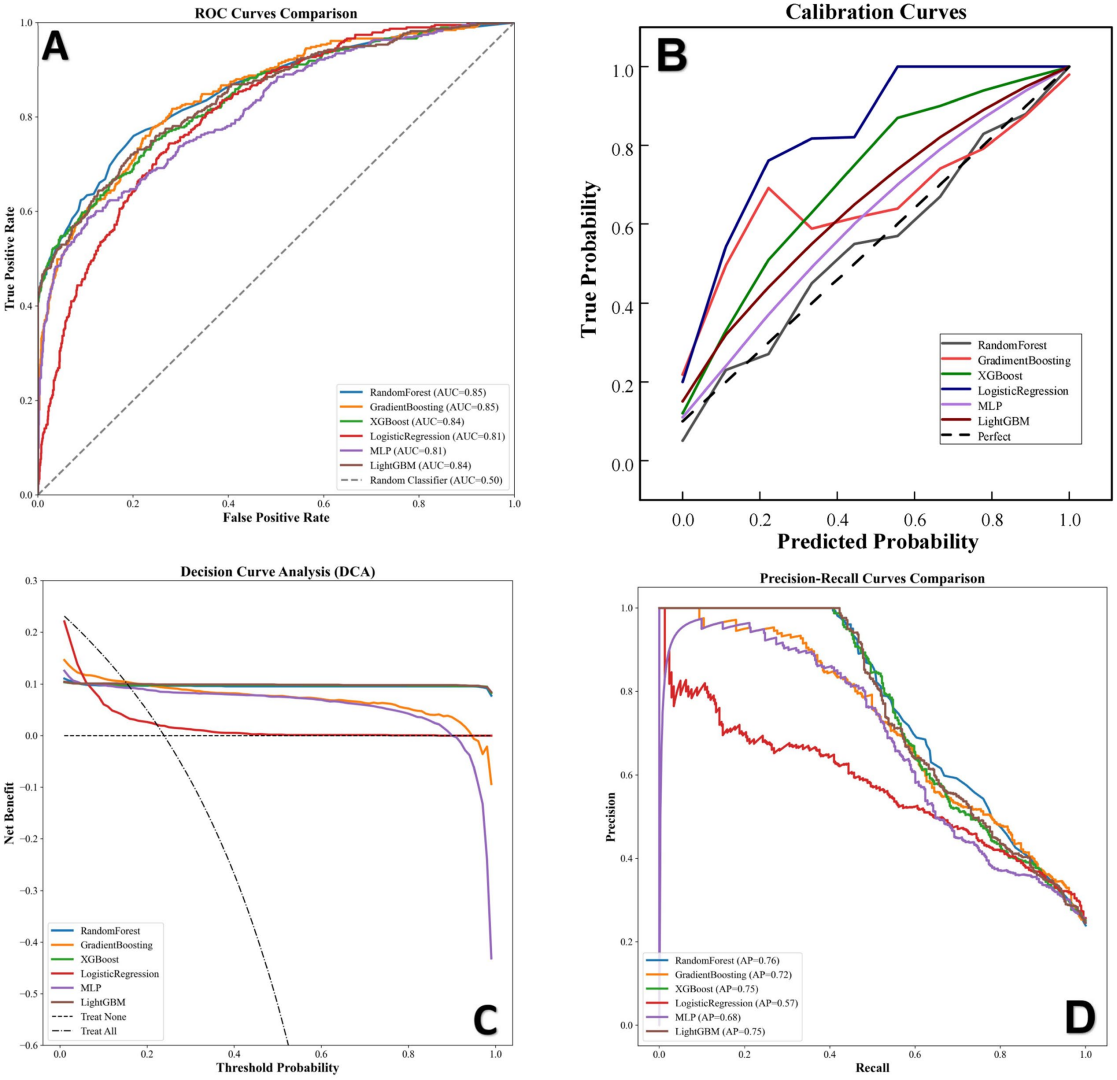
External validation performance of the Random Forest model. eICU-CRD database **(A–C)**: **(A)** ROC curve (AUC = 0.76), **(B)** Decision Curve Analysis, and **(C)** PR curve (AP = 0.77). Institutional database **(D–F)**: **(D)** ROC curve (AUC = 0.74), **(E)** Decision Curve Analysis, and **(F)** PR curve (AP = 0.36). ROC, Receiver Operating Characteristic; AUC, Area Under the Curve; DCA, Decision Curve Analysis; PR, Precision-Recall; AP, Average Precision.

**Figure 4:**
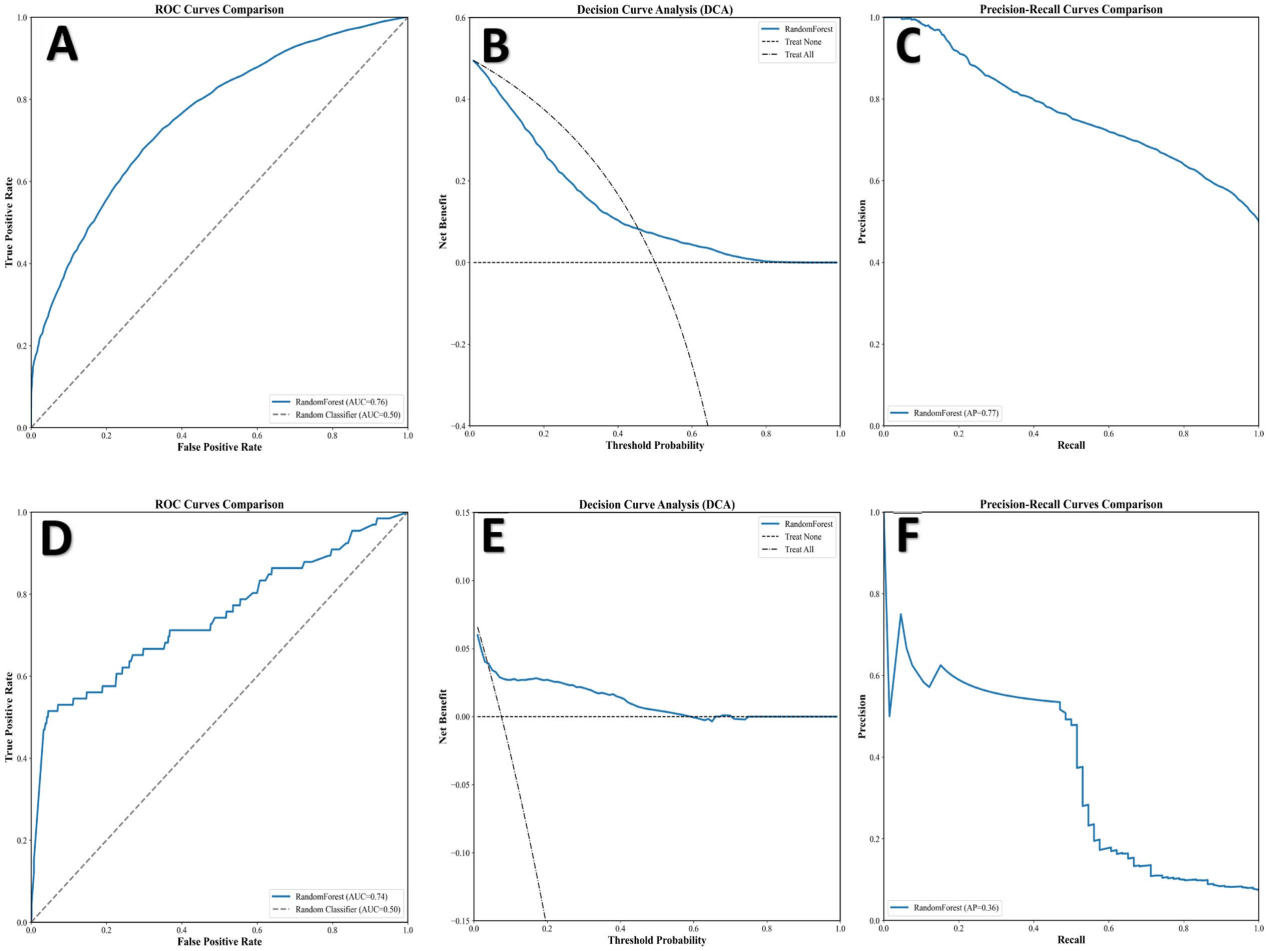
Performance of six trained machine learning models evaluated on the internal validation set. **(A)** Comparison of Receiver Operating Characteristic (ROC) curves. **(B)** Calibration curves. **(C)** Decision Curve Analysis (DCA). **(D)** Comparison of Precision-Recall (PR) curves. AUC, Area Under the Curve; DCA, Decision Curve Analysis; AP, Average Precision; MLP, Multi-Layer Perceptron; XGBoost, eXtreme Gradient Boosting; LightGBM, Light Gradient Boosting Machine.

The dataset was randomly divided into a training set (n = 5,180) and an internal test set (n = 1,295) at a 8:2 ratio. Then, we developed and validated six machine learning (ML) models aimed at identifying the risk of NOAF in critically ill patients. The discriminative performance of these models was comprehensively assessed on the test set, with their ROC curves presented in Figure 3. The predictive performance of the six models was assessed using the AUC of the ROC curve, as shown in Figure 4A. All six models demonstrated robust predictive capabilities, achieving AUCs ranging from 0.808 to 0.855. Notably, the Random Forest model performed best with an AUC of 0.855. It was followed in performance by Gradient Boosting (0.847), LightGBM (0.845), XGBoost (0.842), Multilayer Perceptron (0.814), and Logistic Regression (0.808) Table 2.

In addition to ROC analysis, we further evaluated the models’ performance through calibration curves, Decision Curve Analysis (DCA), and Precision-Recall (PR) curves to assess their reliability and practical value.

The calibration of the models, which measures the agreement between predicted probabilities and actual observed outcomes, is shown in Figure 4B. Visual inspection reveals that the Random Forest model demonstrated the most optimal calibration. To robustly quantify this calibration performance and address the recognized limitations of the Hosmer-Lemeshow test in large datasets, we further computed the calibration slope and intercept. For the optimal Random Forest model, quantitative analysis yielded a calibration slope of 0.902 and a calibration intercept of 0.112.

To evaluate the clinical usefulness of the models, we performed a DCA, presented in Figure 4C. The analysis revealed that all six models provided a significant and positive net benefit across nearly the entire range of threshold probabilities (from 0.05 to 0.95). This result strongly indicates that using any of these models for decision-making is superior to treating all patients or no patients. The ensemble models, particularly Random Forest, consistently showed a marginally higher net benefit, further supporting their value in a practical setting.

The Precision-Recall (PR) curves, which are especially informative for imbalanced datasets, are compared in Figure 4D. The Average Precision (AP), equivalent to the area under the PR curve, was calculated for each model. The Random Forest model once again achieved the highest performance with an AP of 0.76. It was closely followed by the XGBoost and LightGBM models, both with an AP of 0.75. The Gradient Boosting model also performed well with an AP of 0.72. The MLP (AP = 0.68) and Logistic Regression (AP = 0.57) models showed lower performance on this metric. The high AP scores of the ensemble models confirm their robust ability to identify true positive cases without a high number of false positives.

Therefore, after a holistic assessment focusing on discrimination (AUC and AP) and, most importantly, clinical utility (DCA), the Random Forest model was selected as the optimal model. Its unparalleled discriminative power (AUC = 0.855, AP = 0.76) and superior net benefit established it as the most robust and clinically valuable tool among the candidates.

### 3.4 External validation performance

To assess generalizability, we applied the selected Random Forest model to two external validation cohorts. In the public eICU-CRD, the model maintained good performance with an AUC of 0.76 (Figure 3A) and an AP of 0.77 (Figure 3C), while the DCA confirmed its clinical utility by showing a positive net benefit (Figure 3B). Subsequently, in our in-house institutional dataset, it achieved an AUC of 0.74 (Figure 3D) and an AP of 0.36 (Figure 3F), with the DCA again indicating a net benefit (Figure 3E). These results confirm the model’s robust discrimination across different datasets and support its potential for clinical transferability.

### 3.5 Model interpretability and clinical application

**Figure 5:**
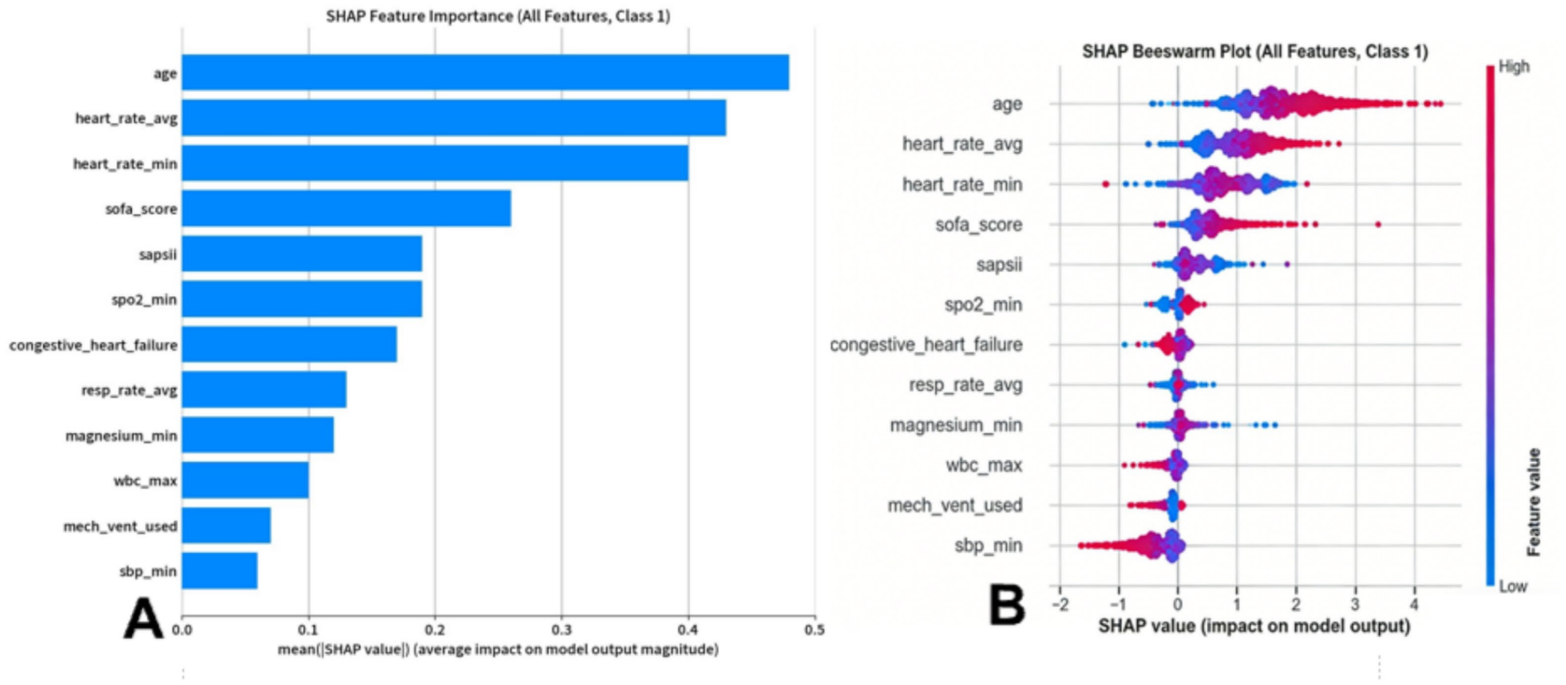
SHAP analysis of feature importance. **(A)** Features ranked by their mean absolute SHAP value. **(B)** SHAP summary plot illustrating the positive or negative impact of features on the model’s prediction.

**Figure 6:**
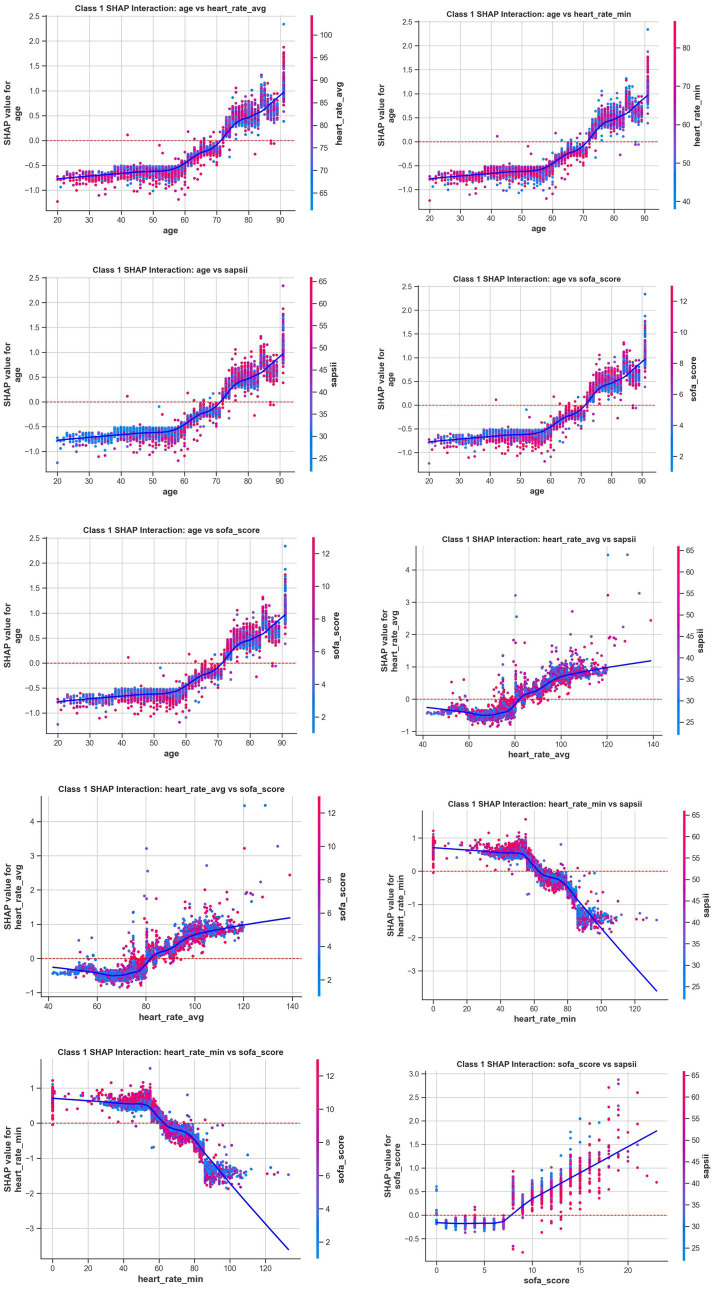
SHAP interaction dependence plots for top-ranking features. Each plot shows the SHAP value of a feature (y-axis) against its value (x-axis), colored by the value of a second, interacting feature. This visualizes how the impact of one feature changes based on the level of another.

To enhance the interpretability of our optimal Random Forest model, we employed the SHAP (SHapley Additive exPlanations) methodology. The analysis identified age, average heart rate (heart_rate_avg), minimum heart rate (heart_rate_min), SOFA score, and SAPS II score as the five most impactful predictors driving the model’s output, as illustrated in the feature importance plot (Figure 5A). The SHAP summary plot (Figure 5B) revealed that higher values for age, average heart rate, SOFA score, and SAPS II score are associated with an increased risk prediction (positive SHAP values). Furthermore, the SHAP interaction plots (Figure 6) revealed significant synergistic effects among top predictors, where the risk contribution of advanced age was significantly amplified in patients with concurrently high SOFA or SAPS II scores.

## 4 Discussion

To address the challenge of predicting NOAF in critically ill patients with CKD, we developed a machine learning model. This multi-center retrospective study utilized the large MIMIC-IV database for model training and internal validation. Crucially, the model’s real-world generalizability was rigorously assessed using two diverse external validation cohorts: the large-scale eICU-CRD and a unique, single-center cohort from our own institution. The Random Forest algorithm outperformed five other models, achieving an AUC of 0.855 in internal validation. It yielded an AUC of 0.74 on our local patient data, confirming its applicability in a specific clinical setting, and an AUC of 0.76 on the eICU-CRD. While these results demonstrate the model’s retained predictive value, the observed attenuation in performance compared to the internal cohort warrants critical evaluation. To translate this predictive power into clinical insights, we utilized SHAP analysis to elucidate the key factors driving the model’s predictions.

The incidence of atrial fibrillation in the ICU is very high, with one in six patients developing the condition (13). Atrial fibrillation is often accompanied by hemodynamic instability, leading to prolonged mechanical ventilation, increased ICU length of stay, and even worsening of cardiac and renal dysfunction (5). Routine electrocardiography misses diagnoses because most episodes are transient and asymptomatic. Early ICU monitoring of atrial fibrillation identifies high-risk patients for intervention implementation and outcome improvement, yet current guidelines and research rarely address specific NOAF predictive management in ICU settings, especially for highly vulnerable populations like those with CKD.

For these patients, the clinical significance of early screening for NOAF has become increasingly prominent. CKD patients are already in a high-risk cardiovascular environment (14), and the occurrence of AF further disrupts the balance of hemodynamics, significantly increasing the probability of thrombosis and embolic events (15). Timely and accurate identification of the presence of AF is crucial for preventing fatal complications and reducing mortality. Addressing this challenge requires more than general prediction tools, it necessitates a model specifically tailored to this vulnerable population.

While prediction models for NOAF have been developed for both general ICU populations and individuals with CKD, a significant gap remains: there is no tool that effectively addresses the acute risk of NOAF specifically within the critically ill CKD population.

First, existing general ICU models suffer from several critical limitations. Traditional models primarily focus on chronic clinical risk factors such as age, blood pressure, and diabetes (16), often overlooking acute, ICU-specific triggers—like persistent infection, inflammation, hemodynamic instability, and mechanical ventilation—that are crucial precipitating mechanisms in this setting. Furthermore, the robustness of many models is questionable, as they are typically trained and validated only within single-center or internal cohorts, lacking the independent external validation across diverse populations necessary to ensure broad generalizability (17). Methodological flaws, such as the failure to rigorously exclude patients with a pre-existing history of AF during cohort selection, can also confound results, preventing the accurate identification of true new-onset events (18). These deficiencies underscore the limited clinical applicability of current models. Established prediction tools such as the CHARGE-AF score demonstrate effectiveness in forecasting atrial fibrillation within general populations by analyzing electronic health records and incorporating variables like age, race, and comorbidities to estimate five-year risk (19), yet these models prove unsuitable for direct application in intensive care settings where patient acuity, dynamic physiological changes, and distinct clinical trajectories necessitate specialized predictive approaches. These prediction tools demonstrate limited applicability in intensive care settings, as they were developed for stable outpatient populations rather than addressing the rapidly changing clinical conditions and distinct patient characteristics found in critical care units.

Second, models created for the general CKD population are equally ill-suited for critical care. The model by Zelnick et al. (20) serves as a prime example. Developed using a stable outpatient cohort (CRIC), it predicts long-term risk over years, a stark contrast to the immediate, short-term risk assessment vital in the ICU. Moreover, by defining the outcome as “hospitalization for AF,” it systematically overlooks the transient and asymptomatic episodes that continuous ICU monitoring is designed to detect. Ultimately, this model achieved only moderate discrimination (C-index≈0.72) and was never externally validated.

In conclusion, there is currently a lack of accurate and reliable general NOAF prediction models for the ICU, and even more so, a lack of a predictive tool specifically tailored for the special population of critically ill patients with CKD. This study aims to fill this critical gap.

To overcome the limitations of traditional prediction tools, machine learning has emerged as a crucial technology. It possesses powerful computing capabilities and can handle large-scale, heterogeneous data from sources such as electronic medical records (EMRs) with remarkable adaptability. Unlike models that rely on preconceived assumptions, machine learning models directly learn from the data, enabling them to decipher the complex interrelationships within highly variable patient information. This ability to construct more accurate and robust clinical prediction models offers a promising path for better predicting challenging clinical scenarios, such as NOAF in critically ill patients with CKD, and improving decision support systems for clinicians and researchers (21).

In our comparative analysis of six machine learning algorithms, the Random Forest model yielded the highest predictive performance. The Random Forest algorithm is a powerful ensemble learning method. Its core mechanism involves the construction of numerous decision trees during the training process. The advantage of this method lies in integrating two key techniques: one is Bootstrap Aggregating, which creates diverse training data subsets for each tree; the other is random feature selection during the splitting of each node. This approach effectively reduces the correlation between different decision trees, thereby significantly reducing the variance of the model and enabling it to have a high ability to resist overfitting. In recent years, prediction models based on Random Forest have become one of the cornerstones in the medical field, performing well in various scenarios, including disease risk prediction (22), patient outcome classification (23), and biomarker discovery (24).

SHAP is a crucial framework for interpreting machine learning models that precisely quantifies the contribution of each feature to individual predictions, thereby revealing the internal decision-making logic of models and enhancing the transparency and interpretability of their prediction processes. Topping the list of predictors was age. Age is a significant independent risk factor. A survey of 15,000 US community-dwelling residents demonstrated that the annual incidence of AF increases exponentially with age: approximately 0.3 cases per 1,000 person-years in the 45–49 age group, 7.5 cases per 1,000 person-years in the 70–74 age group, and over 15 cases per 1,000 person-years in the 80–84 age group (25). Elderly patients with atrial fibrillation pose unique challenges for clinical management, largely due to their prevalent frailty and comorbid conditions. Consequently, early and precise identification of this high-risk population has become central to improving their prognosis (26). In our SHAP analysis, heart rate metrics, specifically the average and minimum heart rate, emerged as the second most influential factor in predicting NOAF. This finding is clinically intuitive, as alterations in heart rate dynamics often reflect autonomic nervous system dysregulation, a well-established pathophysiological driver of atrial arrhythmogenesis. The model’s reliance on this feature under scores its ability to capture critical, early-stage physiological instability preceding the onset of AF (27). Following heart rate, the SHAP analysis identified SOFA and SAPS II scores as the next key influencing factors. This finding underscores the close association between the overall severity of a patient’s condition and the risk of NOAF. As these scores summarize the extent of organ dysfunction and physiological stress, their significance in the model confirms that what drives the risk of arrhythmia in this vulnerable population is the patient’s overall critical condition, rather than isolated physiological parameters (28). In addition to the overall disease severity score, the model also identified hypoxemia (represented by SpO_2__min) and respiratory compensation (represented by resp_rate_avg) as important predictors. This finding is in line with clinical practice, where the deterioration of respiratory function and the subsequent systemic hypoxia are key triggers for the development of NOAF in critically ill patients (29). Hypoxia can promote the occurrence of arrhythmias by directly affecting the electrophysiological properties of atrial muscle and indirectly exacerbating the imbalance of the autonomic nervous system (30). The model’s sensitivity to these real-time vital signs highlights its ability to capture acute physiological deterioration. The model identified congestive heart failure as a strong predictor, which is of great significance. This validates that our model is in line with existing clinical knowledge, as heart failure is widely recognized as a definite precursor of atrial fibrillation due to the resulting atrial remodeling (31). In the CKD population we studied, this association was significantly amplified because the interaction between heart and kidney dysfunction (i.e., heart-kidney syndrome) is very common (32). By capturing this crucial comorbidity, the model demonstrated its ability to identify the inherent complex risks of this specific patient group. In addition, the use of mechanical ventilation was also identified as a relevant feature. This might reflect the cumulative effect of multiple factors: it is both a marker of the severity of the disease, and may independently contribute to the risk of atrial fibrillation through its direct impact on hemodynamics and intrathoracic pressure (33). In addition to the top-ranked predictors, the model also assigns importance to other clinically intuitive factors, although to a lesser extent. These factors include markers of systemic inflammation and hemodynamic instability (such as wbc_max and sbp_min) (34), as well as electrolyte imbalances such as hypomagnesemia (magnesium_min) (35). Overall, the inclusion of these features once again confirms this principle: in such circumstances, NOAF is a multi-factor process, the result of the accumulation of various stress factors, rather than a single trigger. The model is able to capture these nuances, further validating its clinical applicability. Although data on height and NT-proBNP were not obtained, the importance of these variables as potential influencing factors for atrial fibrillation cannot be ignored.

The model interpretability analysis conducted using SHAP revealed the hierarchical risk factors for the development of NOAF in critically ill patients with CKD. The strongest predictors identified by the model were advanced age, elevated average and minimum heart rates, and a higher disease severity score. Following these were key physiological disorders, such as hypoxemia and baseline comorbidities like a history of congestive heart failure, which also significantly increased the risk. Therefore, when patients exhibit a combination of these demographic, hemodynamic, and pathophysiological risk markers, clinicians should remain highly vigilant about impending atrial fibrillation.

To maximize clinical applicability, this model is activated 24 h after the patient is admitted to the ICU. This time window can both prevent drastic fluctuations in physiological indicators upon the patient’s initial admission and enable judgments based on more stable clinical data. In practical applications, when the model identifies a high risk of NOAF in the patient, a specific preventive workflow will be triggered. Clinicians can actively enhance continuous electrocardiogram monitoring to detect early or asymptomatic episodes of atrial fibrillation as early as possible. Additionally, based on the results of SHAP analysis, doctors can promptly screen and correct reversible causes (such as electrolyte imbalances, hypoxemia), or consider preventive treatment measures for the highest-risk patients.

Although the model maintained an acceptable discriminatory ability in external validation, the observed performance decline in the eICU-CRD (with the internal AUC dropping from 0.855 to 0.76 externally) is an expected yet crucial phenomenon, highlighting the universal challenges faced by model transferability. We should not simply describe this as a broad generalization ability but must critically examine several real-world factors that led to this performance degradation. Firstly, the cohort differences are significant. MIMIC-IV reflects a specific patient population from a single academic medical center, while eICU-CRD aggregates data from over 200 different hospitals across the United States. This in itself brings greater variability in case mix, baseline comorbidities, and care protocols. Specifically, the MIMIC cohort includes a higher proportion of advanced/substantial CKD cases, which is in line with its status as a top academic referral center; while the eICU includes many community hospitals, and the CKD patient population is more diverse and the conditions are relatively milder. This results in the two databases being incompletely comparable, and also explains why models trained on severe CKD perform poorly when applied to the general CKD population. Secondly, despite the strict mapping, noise is inevitably introduced when coordinating variables across different electronic health record (EHR) systems. Differences in record frequency, laboratory testing methods, and mechanisms for handling missing data can all alter the underlying data distribution. Thirdly, the differences in severity scores (such as SOFA and SAPS II, which our SHAP analysis has identified as key features) are actually unavoidable. The calculation of these scores is highly dependent on the frequency and completeness of clinical documentation, and there are systematic differences in this regard among different institutions within the eICU-CRD network. Finally, the practice differences among various institutions directly affect the model performance. Different ICUs adopt different thresholds in initiating continuous telemetry monitoring, ordering 12-lead electrocardiograms, and handling acute trigger events. All these factors influence the true detection rate of transient NOAF episodes. Therefore, this performance decline is an extremely informative indicator, suggesting that complex models inherently capture the data characteristics of specific institutions and further reinforcing the critical requirement of local re-calibration before clinical deployment.

In addition to the internal predictive performance, addressing the issue of the generalizability of machine learning models across different medical environments is also a crucial task. For a long time, predictive tools in the intensive care field have mainly relied on databases from the United States (such as MIMIC and eICU-CRD), which may limit their generalizability due to inherent regional biases. The successful validation of our model in an independent Chinese intensive care unit cohort (achieving an AUC value of 0.74) represents an important advantage of this study. This non-US validation itself highlights the robustness of the model to changes in different national medical systems and local intensive care practices. There are significant differences in intensive care strategies between the United States and China, including admission criteria for intensive care units, allocation of medical resources, triage thresholds, and baseline patient characteristics. Despite these significant real-world heterogeneity, the core clinical predictive factors identified in our study still maintain their predictive value. This indicates that our final model does not overly rely on algorithm selection or overly fit to specific clinical protocols - these are common flaws in complex machine learning methods - but is based on basic physiological indicators that transcend local differences in intensive care units. Therefore, this enables the expansion of the clinical application scope of our model in non-US regions.

### 4.1 Limitation

When interpreting the results of this study, several key limitations should be taken into account. Firstly, the retrospective study design fundamentally limits our ability to establish robust causal relationships and makes the study susceptible to unmeasured confounding factors and selection bias. The data in EHR come from clinical treatment records rather than being collected for research purposes, which suggests that a prospective study design should be adopted in the future to better control these biases.

Secondly, there is a risk of misclassification in our reliance on administrative records (such as ICD codes). The initial identification of NOAF mainly depends on ICD codes; although we cross-validated these codes through electrocardiogram reports in the external cohort, transient or asymptomatic NOAF episodes are likely to still be underreported. Similarly, defining the CKD cohort through administrative codes rather than continuous biomarker measurements (such as the dynamic trajectory of eGFR changes) may lack sufficient clinical precision, thereby introducing heterogeneity in the baseline population.

Thirdly, as our external analysis indicates, the transferability of the model is significantly limited. The model exhibited performance degradation in both the multi-center eICU-CRD and the non-US (Chinese) cohort, suggesting that differences in ICU clinical practices (such as the threshold setting for continuous electrocardiogram monitoring) and variations in the underlying population characteristics can significantly affect the model’s performance in the real world, thereby restricting the model’s ready applicability and generalization ability.

Finally, the inherent structure of the used database and the presence of data gaps have prevented us from incorporating several well-known predictors, resulting in potential residual confounding. Specifically, due to the lack of unified records, some important variables were not included in the model, including complete structured data on specific medication histories (such as whether the patient had used beta-blockers, anti-arrhythmic drugs, or angiotensin-converting enzyme inhibitors (ACEI) in the past), as well as detailed echocardiographic parameters (such as left atrial volume index or existing structural changes). Due to the inability to include these specific markers, as well as variables with a high rate of missing data like height and NT-proBNP, the actual predictive ability of the model is likely to be limited.

## 5 Conclusion

This study successfully developed and validated a machine learning model for predicting NOAF risk in critically ill patients with CKD. The model demonstrated good performance and shows potential as a reliable clinical tool. Interpretability was enhanced through SHAP analysis, which helps clinicians understand individual patient risk drivers and provides a basis for clinical decision-making and care prioritization. Future research will further validate this model in real-time prospective environments.

## Data Availability

The original contributions presented in the study are included in the article/supplementary material, further inquiries can be directed to the corresponding author/s.
